# Crystal structure of (NH_4_)_2_[Fe^II^_5_(HPO_3_)_6_], a new open-framework phosphite

**DOI:** 10.1107/S1600536814021783

**Published:** 2014-10-08

**Authors:** Teresa Berrocal, Jose Luis Mesa, Edurne Larrea, Juan Manuel Arrieta

**Affiliations:** a Dpto. de Química Inorgánica, Facultad de Ciencia y Tecnología, Universidad del País Vasco, UPV/EHU, 48080 Leioa, Spain; b Dpto. Mineralogía y Petrología, Facultad Ciencia y Tecnología, Universidad del País Vasco, UPV/EHU, 48080 Leioa, Spain

**Keywords:** crystal structure, mild hydro­thermal synthesis, iron phosphite, IR spectroscopy

## Abstract

Di­ammonium hexa­phosphito­penta­ferrate(II), (NH_4_)_2_[Fe_5_(HPO_3_)_6_], was synthesized under mild hydro­thermal conditions and autogeneous pressure, yielding twinned crystals. The crystal structure exhibits an [Fe^II^_5_(HPO_3_)_6_]^2−^ open framework with NH_4_^+^ groups as counter-cations. The anionic skeleton is based on (001) sheets of [FeO_6_] octa­hedra (one with point-group symmetry 3.. and one with .2.) linked along [001] through [HPO_3_]^2−^ oxoanions. Each sheet is constructed from 12-membered rings of edge-sharing [FeO_6_] octa­hedra, giving rise to channels with a radius of *ca* 3.1 Å in which the disordered NH_4_^+^ cations are located. The IR spectrum shows vibrational bands typical for phosphite and ammonium groups.

## Abstract 2

(NH_4_)_2_[Fe^II^_2_(HPO_3_)_6_] exhibits an open-framework structure with channels in which disordered ammonium cations are situated.

## Chemical context   

Research in the area of solids exhibiting open-framework structures continues to be exciting because of their numerous potential applications (Barrer, 1982 ▶; Hagrman *et al.*, 1999 ▶). Prior to the early 1980s when nanoporous aluminium phosphates were first reported by Flanigen and co-workers, aluminosilicate-based zeolites (Wilson *et al.*, 1982 ▶) and closely related systems represented the predominant class of mat­erials with open-framework structures. In the me­antime, a plethora of activities and efforts have been undertaken for the synthesis of numerous other compounds with open-framework structures of different dimensionalities (Yu & Xu, 2006 ▶).

Recently a new ammonium iron phosphite, (NH_4_)[Fe(HPO_3_)_2_], has been reported (Hamchaoui *et al.*, 2013 ▶) that consists of [Fe^III^(HPO_3_)_2_]^−^ layers formed by [FeO_6_] octa­hedra inter­connected by [HPO_3_]^2−^ oxoanions. The ammonium counter-cations are located in the inter­layer space. Here we report on synthesis and the crystal structure of another ammonium iron phosphite, (NH_4_)_2_[Fe_5_(HPO_3_)_6_], in which iron exhibits oxidation state +II.

## Structural commentary   

**Figure 1:**
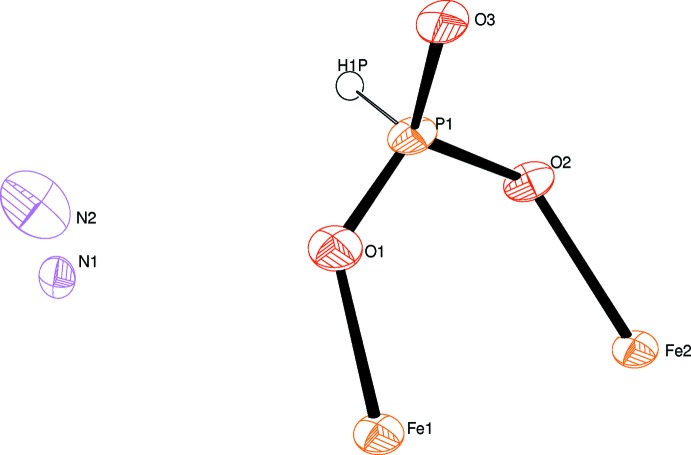
The asymmetric unit of (NH_4_)_2_[Fe^II^_5_(HPO_3_)_6_], with displacement parameters drawn at the 50% probability level.

Tha asymmetric unit of (NH_4_)_2_[Fe_5_(HPO_3_)_6_] is displayed in Fig. 1 ▶. The crystal structure of the title compound contains [FeO_6_] octa­hedra linked *via* edge-sharing into sheets parallel to (001). These sheets consist of 12-membered rings whereby each ring is formed by six [Fe(1)O_6_] octa­hedra and six [Fe(2)O_6_] octa­hedra. The iron(II) ions occupy two different special positions (6*f* and 4*d*) with site symmetries of .2. and 3.., respectively. In one of the FeO_6_ octa­hedra (Fe1), the Fe—O bond lengths range from 2.030 (2) to 2.217 (3) Å while in the [Fe(2)O_6_] octa­hedron a more uniform bond-length distribution from 2.138 (3) to 2.140 (3) Å is observed. The bond angles of the two [FeO_6_] octa­hedra range between 76.48 (10) and 103.18 (9)° for the *cis*- and between 163.65 (12) and 178.24 (17)° for the *trans*-angles.

**Figure 2:**
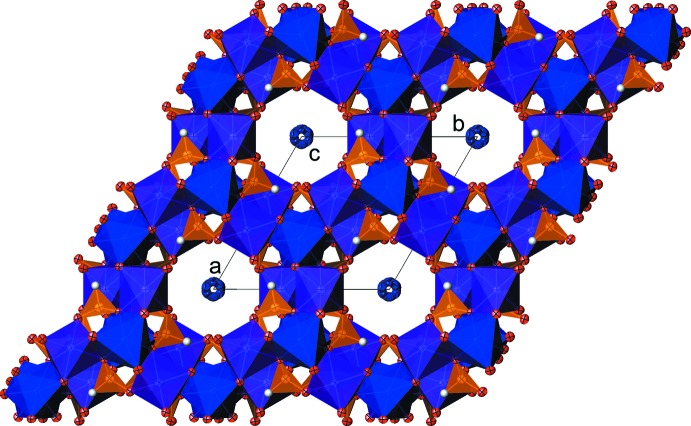
The crystal structure of (NH_4_)_2_[Fe^II^_5_(HPO_3_)_6_] in polyhedral representation, in a projection along [001]. Displacement parameters are drawn at the 50% probability level.

The iron oxide sheets are linked through phosphite groups in which six anions share the most inter­ior oxygen atoms of each ring (Fig. 2 ▶), forming 12-membered channels along [001] with a radius of about 3.1 Å. The phospho­rus(III) atom of the complex oxoanion is located on a general position of this space group. The P—O bond lengths of the anion range from 1.514 (3) to 1.538 (3) Å, and the P—H distance is 1.28 (5) Å, with O—P—O bond angles from 110.28 (17) to 114.29 (17)°.

## Supra­molecular features   

The ammonium cations are located in the 12-membered channels of the framework structure. Although no hydrogen atoms of the cations could be located due to the positional disorder, N⋯O contacts of 2.67 (6), 2.85 (7), 2.87 (8) and 2.98 (6) Å between the cations and the O atoms of the anions suggest hydrogen-bonding inter­actions of medium strength. The H atom of the [HPO_3_]^2−^ anion shows a distance of 2.51 (3) Å to atom O1 [P—H⋯O angle 116.8 (14)°] and seems not to be part of relevant hydrogen-bonding inter­actions.

## Synthesis and characterization   

(NH_4_)_2_[Fe^II^_5_(HPO_3_)_6_] was synthesized under mild hydro­thermal conditions and autogeneous pressure (10–20 bar at 343 K). The reaction mixture was prepared from 30 ml water, 2 ml of phospho­rous acid, 0.17 mmol of NH_4_OH and 0.37 mmol of FeCl_3_. The mixture had a pH value of ≃ 6.0. The reaction mixture was sealed in a polytetra­fluoro­ethyl­ene (PTFE)-lined steel pressure vessel, which was maintained at 343 K for five days. This procedure apparently caused reduction of iron(III) to iron(II) and led to the formation of single crystals of the title compound with a dark-green colour. All crystals appeared to be twinned. The presence of ammonium cations in the title compound was confirmed by infra-red spectroscopy, showing bands at 3190 and 1450 cm^−1^. Characteristic bands of the phosphite P—H group were also observed at 2510 and 1050 cm^−1^ (Nakamoto, 1997 ▶).

## Refinement   

Crystal data, data collection and structure refinement details are summarized in Table 1 ▶. The title crystal was confirmed to be twinned by merohedry using the TwinRotMap option in *PLATON* (Spek, 2009 ▶). The twin element is a 180°-rotation around the <1
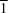
0> direction, or any other equivalent representations of the coset decomposition of the 6/*mmm* holohedry under crystal class 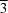 *m*1. The twin law (0
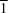
0/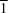
00/00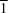) was used during the refinements, and the twin volume of the second component refined to a value of 0.079 (1)%.

The hydrogen atom of the phosphite group was located in a difference map and restrained to be equidistant to the three oxygen atoms of the group, and a fixed isotropic displacement parameter with a value equal to 1.2*U*_eq_ of the parent P atom was assigned.

The ammonium cation is equally disordered around a threefold rotation axis along (00*z*) and was refined with two positions, N1 and N2. The occupancy factors of N1 and N2 were initially freely refined, but since they refined close to the expected value of 1/6, this value was fixed during the last cycles. Because the ellipsoids of these atoms were very elongated, ISOR commands of *SHELXL2014* (Sheldrick, 2008 ▶) were used to achieve more regular displacements. This command restrains the *U*_ij_ components of anisotropically refined atoms to behave approximately isotropically within a standard uncertainty. H atoms belonging to the disordered ammonium atoms were not considered in the final model.

## Supplementary Material

Crystal structure: contains datablock(s) global, I, New_Global_Publ_Block. DOI: 10.1107/S1600536814021783/wm5036sup1.cif

Structure factors: contains datablock(s) I. DOI: 10.1107/S1600536814021783/wm5036Isup2.hkl

CCDC reference: 1027279

Additional supporting information:  crystallographic information; 3D view; checkCIF report

## Figures and Tables

**Figure 3:**
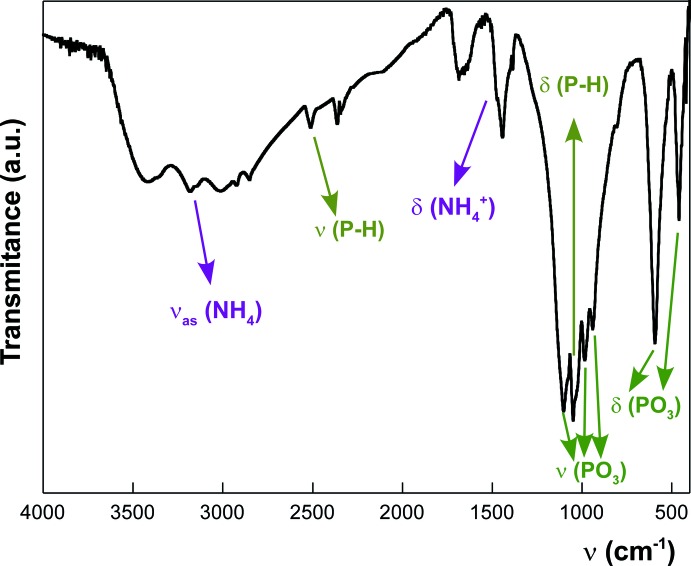
The IR spectrum of (NH_4_)_2_[Fe^II^_5_(HPO_3_)_6_], with partial band assignments.

**Table 1:** Experimental details

Crystal data	
Chemical formula	(NH_4_)_2_[Fe_5_(HPO_3_)_6_]
*M*_r_	795.20
Crystal system, space group	Trigonal, *P* 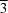 *c*1
Temperature (K)	100
*a*, *c* ()	10.3862(15), 9.2089(14)
*V* (^3^)	860.3(3)
*Z*	2
Radiation type	Mo *K*
(mm^1^)	4.78
Crystal size (mm)	0.18 0.05 0.02

Data collection	
Diffractometer	Agilent SuperNova (single source at offset)
Absorption correction	Gaussian (*CrysAlis PRO*; Agilent, 2014 ▶)
*T*_min_, *T*_max_	0.566, 0.893
No. of measured, independent and observed [*I* > 2(*I*)] reflections	6343, 659, 618
*R*_int_	0.073
(sin /)_max_ (^1^)	0.649

Refinement	
*R*[*F* ^2^ > 2(*F* ^2^)], *wR*(*F* ^2^), *S*	0.033, 0.065, 1.17
No. of reflections	659
No. of parameters	68
No. of restraints	15
H-atom treatment	Only H-atom coordinates refined
_max_, _min_ (e ^3^)	0.53, 0.76

Computer programs: *CrysAlis PRO* (Agilent, 2014 ▶), *SUPERFLIP* (Palatinus Chapuis, 2007 ▶), *SHELXL2014* (Sheldrick, 2008 ▶), *DIAMOND* (Brandenburg, 2010 ▶) and *OLEX2* (Dolomanov *et al.*, 2009 ▶).

## supplementary crystallographic information

### Crystal data

**Table:**

(NH_4_)_2_[Fe_5_(HPO_3_)_6_]	*D*_x_ = 3.070 Mg m^−^^3^
*M**_r_* = 795.20	Mo *K*α radiation, λ = 0.71073 Å
Trigonal, *P*3*c*1	Cell parameters from 2414 reflections
*a* = 10.3862 (15) Å	θ = 2.3–28.3°
*c* = 9.2089 (14) Å	µ = 4.78 mm^−^^1^
*V* = 860.3 (3) Å^3^	*T* = 100 K
*Z* = 2	Acicular, dark green
*F*(000) = 784	0.18 × 0.05 × 0.02 mm

### Data collection

**Table:**

Agilent SuperNova (single source at offset) diffractometer	659 independent reflections
Radiation source: SuperNova (Mo) X-ray Source	618 reflections with *I* > 2σ(*I*)
Mirror monochromator	*R*_int_ = 0.073
Detector resolution: 16.2439 pixels mm^-1^	θ_max_ = 27.5°, θ_min_ = 2.2°
ω scans	*h* = −13→11
Absorption correction: gaussian (*CrysAlis PRO*; Agilent, 2014)	*k* = −10→13
*T*_min_ = 0.566, *T*_max_ = 0.893	*l* = −11→11
6343 measured reflections	

### Refinement

**Table:**

Refinement on *F*^2^	Primary atom site location: structure-invariant direct methods
Least-squares matrix: full	Secondary atom site location: difference Fourier map
*R*[*F*^2^ > 2σ(*F*^2^)] = 0.033	Hydrogen site location: difference Fourier map
*wR*(*F*^2^) = 0.065	Only H-atom coordinates refined
*S* = 1.17	*w* = 1/[σ^2^(*F*_o_^2^) + (0.0126*P*)^2^ + 2.2823*P*] where *P* = (*F*_o_^2^ + 2*F*_c_^2^)/3
659 reflections	(Δ/σ)_max_ < 0.001
68 parameters	Δρ_max_ = 0.53 e Å^−^^3^
15 restraints	Δρ_min_ = −0.76 e Å^−^^3^

### Special details

**Table:**

Geometry. All e.s.d.'s (except the e.s.d. in the dihedral angle between two l.s. planes) are estimated using the full covariance matrix. The cell e.s.d.'s are taken into account individually in the estimation of e.s.d.'s in distances, angles and torsion angles; correlations between e.s.d.'s in cell parameters are only used when they are defined by crystal symmetry. An approximate (isotropic) treatment of cell e.s.d.'s is used for estimating e.s.d.'s involving l.s. planes.
Refinement. Refinement of *F*^2^ against ALL reflections. The weighted *R*-factor *wR* and goodness of fit *S* are based on *F*^2^, conventional *R*-factors *R* are based on *F*, with *F* set to zero for negative *F*^2^. The threshold expression of *F*^2^ > 2σ(*F*^2^) is used only for calculating *R*-factors(gt) *etc*. and is not relevant to the choice of reflections for refinement. *R*-factors based on *F*^2^ are statistically about twice as large as those based on *F*, and *R*- factors based on ALL data will be even larger.

### Fractional atomic coordinates and isotropic or equivalent isotropic displacement parameters (Å^2^)

**Table:**

	*x*	*y*	*z*	*U*_iso_*/*U*_eq_	Occ. (<1)
Fe1	0.61330 (9)	0.0000	0.2500	0.0209 (2)	
Fe2	0.6667	0.3333	0.33453 (10)	0.0202 (3)	
P1	0.88002 (13)	0.29352 (13)	0.09044 (10)	0.0224 (3)	
H1P	1.020 (5)	0.343 (3)	0.091 (2)	0.027*	
O2	0.8512 (3)	0.3935 (3)	0.1927 (3)	0.0234 (7)	
O3	0.8404 (3)	0.3102 (4)	−0.0665 (3)	0.0243 (7)	
O1	0.8063 (4)	0.1345 (4)	0.1440 (3)	0.0284 (8)	
N2	0.970 (5)	−0.047 (3)	0.059 (2)	0.045 (9)	0.1667
N1	1.024 (5)	−0.015 (6)	0.2033 (17)	0.020 (7)	0.1667

### Atomic displacement parameters (Å^2^)

**Table:**

	*U*^11^	*U*^22^	*U*^33^	*U*^12^	*U*^13^	*U*^23^
Fe1	0.0286 (4)	0.0250 (5)	0.0080 (4)	0.0125 (2)	0.00013 (16)	0.0003 (3)
Fe2	0.0264 (4)	0.0264 (4)	0.0077 (4)	0.01319 (19)	0.000	0.000
P1	0.0278 (6)	0.0323 (7)	0.0066 (4)	0.0148 (5)	−0.0011 (4)	−0.0018 (4)
O2	0.0265 (17)	0.0314 (17)	0.0088 (12)	0.0119 (14)	−0.0013 (11)	−0.0049 (12)
O3	0.0330 (18)	0.0366 (18)	0.0062 (11)	0.0195 (15)	−0.0043 (11)	−0.0037 (12)
O1	0.041 (2)	0.0311 (18)	0.0123 (13)	0.0176 (16)	−0.0007 (13)	−0.0011 (13)
N2	0.036 (17)	0.040 (17)	0.051 (11)	0.014 (13)	−0.015 (15)	0.004 (10)
N1	0.012 (17)	0.011 (15)	0.030 (8)	0.002 (7)	0.002 (9)	−0.002 (10)

### Geometric parameters (Å, º)

**Table:**

Fe1—O2^i^	2.086 (3)	Fe2—O3^vii^	2.140 (3)
Fe1—O2^ii^	2.086 (3)	Fe2—O3^viii^	2.140 (3)
Fe1—O3^iii^	2.217 (3)	P1—O2	1.538 (3)
Fe1—O3^iv^	2.217 (3)	P1—O3	1.536 (3)
Fe1—O1	2.030 (3)	P1—O1	1.514 (3)
Fe1—O1^v^	2.030 (3)	O2—Fe1^vi^	2.086 (3)
Fe2—O2	2.138 (3)	O3—Fe1^ix^	2.217 (3)
Fe2—O2^ii^	2.138 (3)	O3—Fe2^x^	2.140 (3)
Fe2—O2^vi^	2.138 (3)	P1—H1P	1.28 (5)
Fe2—O3^iii^	2.140 (3)		
			
O2^i^—Fe1—O2^ii^	87.23 (17)	O2—Fe2—O3^vii^	163.65 (11)
O2^ii^—Fe1—O3^iv^	102.21 (11)	O2—Fe2—O3^viii^	77.08 (11)
O2^ii^—Fe1—O3^iii^	76.48 (10)	O2^vi^—Fe2—O3^vii^	77.08 (11)
O2^i^—Fe1—O3^iv^	76.48 (10)	O2^ii^—Fe2—O3^viii^	163.65 (11)
O2^i^—Fe1—O3^iii^	102.21 (11)	O2^ii^—Fe2—O3^iii^	77.08 (11)
O3^iv^—Fe1—O3^iii^	178.24 (17)	O3^iii^—Fe2—O3^vii^	103.18 (9)
O1^v^—Fe1—O2^ii^	165.86 (10)	O3^iii^—Fe2—O3^viii^	103.18 (9)
O1^v^—Fe1—O2^i^	87.80 (12)	O3^viii^—Fe2—O3^vii^	103.18 (9)
O1—Fe1—O2^ii^	87.80 (12)	O3—P1—O2	110.28 (17)
O1—Fe1—O2^i^	165.86 (10)	O1—P1—O2	111.96 (17)
O1^v^—Fe1—O3^iv^	89.46 (11)	O1—P1—O3	114.29 (17)
O1—Fe1—O3^iii^	89.46 (11)	Fe1^vi^—O2—Fe2	103.34 (12)
O1—Fe1—O3^iv^	91.67 (11)	P1—O2—Fe1^vi^	127.30 (17)
O1^v^—Fe1—O3^iii^	91.67 (11)	P1—O2—Fe2	128.71 (18)
O1^v^—Fe1—O1	99.93 (18)	Fe2^x^—O3—Fe1^ix^	99.01 (10)
O2^ii^—Fe2—O2^vi^	86.57 (11)	P1—O3—Fe1^ix^	124.27 (18)
O2—Fe2—O2^vi^	86.57 (11)	P1—O3—Fe2^x^	134.56 (18)
O2—Fe2—O2^ii^	86.57 (11)	P1—O1—Fe1	133.9 (2)
O2—Fe2—O3^iii^	92.54 (11)	O1—P1—H1P	106.9 (14)
O2^vi^—Fe2—O3^viii^	92.54 (11)	O2—P1—H1P	107.0 (12)
O2^ii^—Fe2—O3^vii^	92.54 (11)	O3—P1—H1P	105.9 (12)
O2^vi^—Fe2—O3^iii^	163.65 (12)		
			
O2—P1—O3—Fe1^ix^	120.4 (2)	O3—P1—O1—Fe1	−91.5 (3)
O2—P1—O3—Fe2^x^	−39.2 (3)	O1—P1—O2—Fe1^vi^	141.7 (2)
O2—P1—O1—Fe1	34.8 (3)	O1—P1—O2—Fe2	−27.5 (3)
O3—P1—O2—Fe1^vi^	−89.9 (2)	O1—P1—O3—Fe1^ix^	−112.4 (2)
O3—P1—O2—Fe2	101.0 (2)	O1—P1—O3—Fe2^x^	88.0 (3)

Symmetry codes: (i) *y*, *x*−1, −*z*+1/2; (ii) −*x*+*y*+1, −*x*+1, *z*; (iii) −*y*+1, −*x*+1, *z*+1/2; (iv) *x*−*y*, *x*−1, −*z*; (v) *x*−*y*, −*y*, −*z*+1/2; (vi) −*y*+1, *x*−*y*, *z*; (vii) −*x*+*y*+1, *y*, *z*+1/2; (viii) *x*, *x*−*y*, *z*+1/2; (ix) *y*+1, −*x*+*y*+1, −*z*; (x) −*y*+1, −*x*+1, *z*−1/2.
